# Synthesis and characterization of keratinase laden green synthesized silver nanoparticles for valorization of feather keratin

**DOI:** 10.1038/s41598-023-38721-6

**Published:** 2023-07-18

**Authors:** Isha Sharma, Pranshi Gupta, Naveen Kango

**Affiliations:** Department of Microbiology, Dr. Harisingh Gour Vishwavidyalaya (A Central University), Sagar, Madhya Pradesh 470003 India

**Keywords:** Microbiology, Environmental sciences

## Abstract

This study focuses on the efficient and cost-effective synthesis of silver nanoparticles (AgNPs) using plant extracts, which have versatile and non-toxic applications. The research objectives include synthesizing AgNPs from readily available plant extracts, optimizing their production and multi scale characterization, along with exploring their use for enzyme immobilization and mitigation of poultry feather waste. Among the plant extracts tested, the flower extract of *Hibiscus rosa-sinensis* (HF) showed the most potential for AgNP synthesis. The synthesis of HF-mediated AgNPs was optimized using response surface methodology (RSM) for efficient and environment friendly production. Additionally, the keratinase enzyme obtained from *Bacillus* sp. NCIM 5802 was covalently linked to AgNPs, forming a keratinase nanocomplex (KNC) whose biochemical properties were evaluated. The KNC demonstrated optimal activity at pH 10.0 and 60 °C and it displayed remarkable stability in the presence of various inhibitors, metal ions, surfactants, and detergents. Spectroscopic techniques such as FTIR, UV–visible, and X-ray diffraction (XRD) analysis were employed to investigate the formation of biogenic HF-AgNPs and KNC, confirming the presence of capping and stabilizing agents. The morphological characteristics of the synthesized AgNPs and KNC were determined using transmission electron microscopy (TEM) and particle size analysis. The study highlighted the antimicrobial, dye scavenging, and antioxidant properties of biogenic AgNPs and KNC, demonstrating their potential for various applications. Overall, this research showcases the effectiveness of plant extract-driven green synthesis of AgNPs and the successful development of keratinase-laden nanocomplexes, opening possibilities for their use in immobilizing industrial and commercial enzymes.

## Introduction

Nanotechnology has emerged over the past few years as a multifaceted field in the area of science and technology, combining applications of engineering to that of basic environmental, biological, chemical and physical sciences^1–3^. This technology beholds great promises because of structural and functional endurance of the nanoscale materials along with their practical applications^4^. Richard P. Feynman envisioned the concept of nanotechnology in 1959 in his talk as outlined “There’s plenty of room at the bottom” which opened a new gateway of the world for hi-tech imagination of intense miniaturization^5,6^.

The synthesis of nanoparticles (NPs) can be achieved through physical, chemical, or biological methods, offering precise control over their size, structure, and plasmon properties for diverse applications^2,7,8^. In this context, commercially available nanoparticles have attracted crucial attention in recent years as they possess significant physicochemical characteristics and vast applications. The only drawback of physically and chemically fabricated NPs is their hazardous, toxic, and expensive nature of preparation^9,10^ and the chemicals used in the synthesis of these NPs are directly released to the environment where the effluents are not only hazardous for the atmosphere but also to the living creatures^11–13^.

Therefore, the innovative approach arrives in the framework, for the biogenesis of green nanomaterials, through the natural mechanism to obtain an environment-friendly nanostructured material for environmental remediation^14,15^. Microorganisms, including bacteria, yeast, and fungi, have gained attention as suitable alternatives for biologically receptive approaches in nanoparticle synthesis^16,17^. The application of microorganisms in environmental bioremediation has been known since time immemorial^18^. But in the past few years these distinctive characteristics of microbial resources have been foreseen as nano factories^19,20^. Hence, scientists are investigating various natural nooks to isolate this microbial plethora with exceptional ability to reduce the metal ions and produce NPs^20–22^.

Amongst magnanimous metal NPs, AgNPs have caught the curiosity of scientists for the reason of their colossal pertinence in everyday life in the fields of biomedical applications, environmental remediation, wastewater treatment, cosmetics, production of food and feed materials, coating of medical equipment, and various other products. They also acquire exclusive physicochemical properties such as thermal, optical, and electrical which may possibly be employed in numerous of therapeutics, biosensors, light sensitive devices, molecular pathology, epidemiology, biomedicines, diagnostics, and imaging applications have propelled them to the forefront of cutting-edge research^23–26^. Moreover, it has been observed that the biogenesis provides a facile, stable, and safe fabrication of the desired NPs and exhibits strong antimicrobial properties and by virtue of their exclusive properties they can infiltrate the cell membrane of the pathogenic microbes and prevents the proliferation by disturbing their cell machinery^27,28^. In this context, the exploration of natural resources, including *Hibiscus rosa-sinensis*, a plant known for its vibrant flowers and medicinal properties, has captured the attention of researchers seeking novel and eco-friendly pathways for nanoparticle synthesis. This captivating plant holds immense potential owing to its rich reservoir of bioactive compounds, which can serve as reducing agents and stabilizers in the green synthesis of nanoparticles with diverse applications.

The objective of this research endeavor is to develop an efficient, cost-effective, and environmentally sustainable method for synthesizing silver nanoparticles from plant extract. The current investigation delves into the green synthesis of AgNPs using the flower extract of *H. rosa-sinensis*, with a meticulous optimization process employed for biogenesis. Furthermore, the keratinase from *Bacillus velezensis* NCIM 5802 was covalently immobilized onto glutaraldehyde-activated HF-AgNPs. The comprehensive analysis of the synthesized NPs and KNC was conducted at various scales, shedding light on the fabrication process of AgNPs, and extensive investigations were carried out to unravel the applications and efficacy of the synthesized AgNPs and KNC.

## Materials and methods

### Chemicals and materials

Silver nitrate (AgNO_3_), glutaraldehyde solution (25%), 2,2-diphenyl-1-picrylhydrazyl (DPPH) reagent and keratin azure were purchased from Sigma-Aldrich, India. Microbiological media components were procured from HiMedia^®^, India. Analytical grade chemicals were used in this study with the highest available purity.

### Screening for the biogenic synthesis of silver nanoparticles (AgNPs)

#### Primary screening of plant extracts

Fresh leaves and flowers of different angiosperms such as *Jasminum* (Chameli; JF, JL), *Catharanthus roseus* (Sadabahar; CF, CL), *Cascabela thevetia* (Kaner; CtF, CtL), *Royal poinciana* (Gulmohar; RF, RL), *Hibiscus rosa-sinensis* (Gudhal; HF, HL) and a gymnosperm *Cycas* (Cy) were collected from the University campus of Sagar MP, India (23.8388° N, 78.7378° E) in accordance with institutional guidelines. The collected samples were further processed according to the method described by Khorrami et al.^29^ with certain modifications The samples were washed thrice with tap water to remove any dirt and then were air dried overnight at 28 °C. The plant extract was then prepared by adding 2.5 g of each sample to 100 mL of lukewarm water. These mixtures were stirred for 30 min and the extract was filtered through Whatman filter paper. The clear supernatant so obtained was used further for the biogenesis of AgNPs.

#### Green synthesis of AgNPs

AgNPs were synthesized according to the method described by Choukade et al.^2^ with certain variations. Concisely, 1 mL plant extract was added to 10 mL of 2 mM AgNO_3_ solution in distilled water followed by incubation at 28 °C and the time duration of AgNPs formation by different samples was recorded. The AgNPs from different plant extracts were collected by centrifugation at 12,000*g* for 30 min, washed twice with distilled water and stored at 4 °C.

#### Secondary screening of plant extracts

##### UV–visible spectrum of synthesized AgNPs

To confirm the formation of AgNPs, UV–visible spectroscopy was performed using a Multiskan spectrophotometer (Thermo Fisher Scientific, USA). Surface Plasma Resonance (SPR) of AgNO_3_, plant extract and synthesized AgNPs, samples were analyzed in the 350–700 nm spectral range. All spectra were recorded in appropriately diluted solutions^29^.

##### Antimicrobial assay

Agar well-diffusion assay was employed to evaluate the anti-bacterial potential of synthesized AgNPs as explained by Kanniah et al.^30^ with certain modifications. The anti-bacterial potency of AgNPs was assessed on nutrient agar plates against *Bacillus cereus* and *B. pumilus*. Bacterial suspension (1 × 10^6^ cells) was uniformly spread on the plates and 100 µL of AgNPs suspension was poured on 6 mm wells made on agar surface from sterile corkborer, water was taken as a control. Plates were then incubated at 37 °C for 24 h and the zone of inhibition was measured^31^.

### Optimization of process parameters by response surface methodology

The process parameters for the green synthesis of AgNPs, three independent variables were taken i.e., (A) silver nitrate concentration (mM), (B) plant extract (%), and (C) reaction time (min) were optimized using Design-Expert v13-Stat-Ease software through Central Composite Design (CCD)^4^. Based on the randomized factorial design, a plot of overall 18 experiments were generated that comprised of 5 levels (− α, − 1, 0, + 1, + α) with three variables, and six replica trials of central points. These trials were performed to study the cumulative effects and to determine the best possible combination of factors through the response pattern for biosynthesis. The response of the design was measured in terms of absorbance at 450 nm. The results of the experiments were analyzed through a second-order polynomial equation. Cumulative interactions between the individual variables were characterized by 3D response surface plots on the basis of R^2^ values, lack of fit & F values. The final outcome and fitness of the obtained model was confirmed by ANOVA^32^.

### Keratinase‑nanocomplex (KNC) preparation

#### Surface activation of synthesized nanoparticles

Green AgNPs (1 mg) were activated by immersing in glutaraldehyde solution (1.5% v/v Tris–HCl buffer 50 mM, pH 7.0) for 4 h at 30 °C. The activated nanoparticles (Glu-NPs) were centrifuged at 10,000*g* for 30 min and were washed thrice to remove excess glutaraldehyde from the pellets with the same buffer. Nanoparticles were re-suspended in the Tris–HCl buffer (50 mM, pH 7.0) and were sonicated using 5 × 5 s pulse of 10% amplitude to avoid aggregation (PKS-750F, PCi Analytics, India)^33^.

#### Formation of keratinase-silver nanocomplex (KNC)

The glutaraldehyde activated AgNPs (10 mL) were incubated with 1 mL keratinase (200 U/mL) derived from *B. velezensis* NCIM 5802^34^. The mixture was slowly stirred overnight at 4 °C and keratinase-AgNPs (KNC) were centrifuged at 8000*g* for 20 min, the pellets so obtained were washed thrice with Tris–HCl buffer (50 mM, pH 7.0) to remove any unbound enzyme. KNC formation was confirmed by performing keratinase activity and measuring characteristic SPR peak. The immobilization yield was calculated using following formula:

1 $$ {\mathrm{Immobilization Yield}}= \frac{{\text{E}_\text{f}}}{\text{E}_\text{i}} \times 100$$

where, E_f_ is the keratinase activity after immobilization and E_i_ is the keratinase activity of free enzyme. The immobilized keratinase preparation was stored at 4 °C for further use^2,33^.

### Keratinase assay

Activity of free keratinase and KNC was assessed using keratin azure (substrate) as illustrated earlier^34^. Briefly, 1% w/v of substrate was suspended in 50 mM Tris–HCl buffer (pH 10.0) to which diluted keratinase sample was added and vortexed. The tubes were further, incubated at 60 °C for 1 h. Following incubation, the reaction tubes were centrifuged for 5 min at 10,000*g*, further, the clear supernatant was collected, and the absorbance was recorded at 595 nm against the suitable enzyme blank. Keratinase activity was calculated as previously described^34,35^**.**

### Characterization of keratinase-nanocomplex

#### Effect of pH and temperature

The pH optima of the KNC were determined by incubating them with keratin azure (0.01 g) at different pH (6.0–11.0) using 50 mM of different buffers (Na-citrate, pH 6.0; Tris–HCl, pH 7.0–9.0; glycine–NaOH, pH 10.0 and sodium dihydrogen phosphate, pH 11.0). The effect of temperature on keratinase was assessed by incubating the reaction tubes at various temperatures extending from 30 to 90 °C with keratin azure as a substrate^34^. The enzymatic assay was performed as described above ("Keratinase assay"). Residual keratinase activity of free and immobilized enzyme at different pH and temperature was estimated. The relative keratinase activity was calculated with respect to the suitable enzyme blank as previously described^36,37^.

#### Influence of pH and temperature on enzyme stability

To study the pH stability, KNC was pre-incubated with different buffers (Na-citrate, pH 6.0; Tris–HCl, pH 7.0–9.0; glycine–NaOH, pH 10.0 and sodium dihydrogen phosphate, pH 11.0), respectively, at optimum temperature. The thermostability of the enzyme was assessed by incubating the enzyme samples at different temperatures (50–70 °C) up to 60 min at pH 10.0. Aliquots were drawn out at regular time periods and the relative activity was assessed against the suitable blank.

#### Effect of metal ions, inhibitors, activators, surfactants, and solvents on KNC

To examine the effect of metal ions on KNC, the enzyme activity was studied in the presence of univalent (Na^+^ and K^+^) and bivalent metal ions (Cu^2+^, Ca^2+^, Mg^2+^, Hg^2+^, Zn^2+^, Fe^2+^, & NH_4_^+^). The impact of different inhibitors. The effect of urea, EDTA (ethylenediamine tetraacetic acid), PMSF (phenylmethanesulfonyl fluoride), solvents; methanol, chloroform, and propanol (0.1, 0.5, 1, 2 and 5%) and surfactants such as Triton X-100, and SDS (sodium dodecyl sulfate) was also examined. The keratinase was pre-incubated with the tested metal ions or modulators for 30 min at 30 °C and the relative activity (%) was determined against the suitable blank.

### Multi-scale characterization of AgNPs and KNC

#### Time dependent reaction kinetics

Time dependent reaction kinetics of AgNPs synthesis was studied in the spectral range of 350–700 nm with Multiskan Go spectrophotometer (Thermo Fisher Scientific) from 0 to 60 min, where distilled water and AgNO_3_ solution were taken as a blank, respectively.

#### Surface plasmon resonance (SPR)

SPR of the AgNPs and KNC was recorded in the spectral range of 300–700 nm using Multiskan Go spectrophotometer (Thermo Fisher Scientific), with the appropriately diluted samples^2^.

#### Particle size analysis

Polydispersity index (PDI) and hydrodynamic particle size of synthesized AgNPs and KNC were studied by Dynamic light scattering (DLS) using Nano Plus Zetasizer (Particulate Systems, USA). Samples were sonicated by applying 10 × 10 s pulse of 10% amplitude and were filtered through 0.2 µM syringe filter to remove any impurities^4^.

#### Transmission electron microscopy (TEM)

The size and shape of the synthesized AgNPs and KNC was studied using TEM (FEI Nova NanoTEM™ 450) operating at an accelerating voltage of 200 kV^8,38^. Prior to placing on the carbon-coated copper grid, the samples were air-dried and re-dispersed using ethanol.

#### Fourier-transform infrared (FTIR) spectroscopic analysis of HF-AgNPs and KNC

The presence of capping agents and specific functional groups responsible for the fabrication of the AgNPs and KNC were determined by FTIR analysis^39^. The samples were centrifuged to remove any residues and then, re-suspended in distilled water. The spectra were obtained using an ALPHA II spectrometer, (Bruker) in the wave number region of 4000–500 cm^−1^ with 15 scans at a resolution of 2 cm^−1^ at transmission mode.

#### X-ray diffraction (XRD) analysis

The diffraction pattern of both AgNPs and KNC was analyzed. The crystalline nature and diffraction patterns of the air dried and powdered samples^38^ were recorded using X-ray diffractometer (Bruker D8 ADVANCE, Germany) operated at 30 kV with a 30 mA current and the diffraction spectra were signified by the 2*θ* angle.

### Applications of green synthesized KNC

#### Antibacterial assay

The antibacterial potential of KNCs was evaluated against pathogenic strains *B. licheniformis* and *B. subtilis* maintained on nutrient agar plates. The antimicrobial activity of KNCs was tested using the agar well diffusion method ("Secondary screening of plant extracts") and minimum inhibitory concentration (20–100 µg/mL) was recorded^1,40^. Glutaraldehyde solution was taken as a control.

#### Dye scavenging

500 μL (80 mg/mL) of KNC was added to the methylene blue solution (1% w/v) prepared in distilled water. The samples were vortexed and incubated under the daylight for photoactivation of KNC. The dye without KNC was treated under the same conditions and taken as a control. The reaction was examined for the disappearance of color with respect to the time and the spectra was taken in the range of 400–700 nm. Using the following equation, absorbance at 660 nm was measured to calculate scavenging (%)^8,41^.

2 $$\mathrm{\% }Scavenging= \frac{{A}_{0}-{A}_{1}}{{A}_{0}}\times 100$$

where, A_0_ is the initial concentration (dye solution) and A_1_ is the concentration of dye after photocatalytic degradation.

#### Leaching of DNA and proteins

To evaluate the extent of cellular destruction when treated with KNC, 100 μg/mL of KNC was added in the log phase of *B. subtilis* culture for 6 h at 40 °C. Following the incubation, cells were pelleted down at 8000*g* for 10 min and the clear supernatant was assessed for the degradation of DNA and protein^31^. Untreated bacterial culture was taken as control. Multiskan GO spectrophotometer (Thermo Fisher Scientific), was used for the quantitative assessment of dsDNA and protein at 260 and 280 nm, respectively.

#### Free radical scavenging activity: antioxidant assay

The free radical scavenging activity of KNC was calculated according to the method described by Ibrahim et al.^4^ with some modifications. In brief, 100 μL of KNC was added at different concentrations (20–100 μg/mL) to 100 μL of 1,1-diphenyl-2-picryl-hydrazyl (DPPH) reagent (80 mg/L in ethanol) in a 96-well plate. The mixture was incubated at 30 °C for 1 h in dark. After incubation, absorbance was measured at 517 nm. The free radical scavenging % was calculated using the following equation:

3 $$\mathrm{\% }Free \,radical\, scavenging= \frac{{A}_{c}-{A}_{s}}{{A}_{c}}\times 100$$

where, Ac is the absorbance of control and As is the absorbance of the sample.

#### Biofilm combating assay

Biofilm degradation efficacy of biosynthesized KNC was assessed as explained by Bhatwalkar et al.^42^ with certain alterations. Concisely, for biofilm formation 200 μL of *B. subtilis* was grown in NA medium and incubated at 40 °C for 24 h till mid-log phase reached. After the treatment of bacterial film with KNC at different concentrations (20–100 μg/mL) the plates were again incubated at given conditions. After incubation, films so formed were washed with PBS (phosphate buffered saline) and distilled water. Further, stained with crystal violet (100 µL of 0.1% w/v) and incubated for 10 min at 30 °C. The plates were rinsed thoroughly with distilled water and finally solubilized with 95% (v/v) ethanol and the absorbance at 570 nm was recorded^2^.

### Keratinase‑nanocomplex recycling

KNCs recycling was done as described by Torabizadeh and Mahmoudi^43^ with certain changes. 100 μL of KNC was incubated with 1% w/v feather keratin, obtained from local poultry farm, (pH 10.0) for 20 min at 60 °C. After incubation, KNC were centrifuged, the pellets were washed twice with Glycine–NaOH buffer (50 mM, pH 10.0) and reused for keratin hydrolysis. The process was repeated for eight consecutive cycles. The enzymatic activity was measured as described earlier ("Keratinase assay") and the activity at 0th cycle was considered as 100%.

### Storage stability

The shelf-life of free keratinase and KNC (Glycine–NaOH buffer; 50 mM, pH 10.0) was studied by storing them at 4 °C and at 30 °C for 45 days.

### Data analysis

To ensure accuracy and reliability, all experimental methods and procedures were strictly performed in accordance with standardized operating protocols and good laboratory practices. Throughout the experiments, analytical-grade chemicals with the utmost purity were utilized, and the analysis machines underwent calibration to maintain their precision. To enhance the statistical validity, the experimental setup was replicated three times, and the outcomes were presented as the average value ± standard deviation (SD) and providing a comprehensive representation of the data.

## Results

### Biogenesis of silver nanoparticles: screening of plant extracts

**Figure 1:**
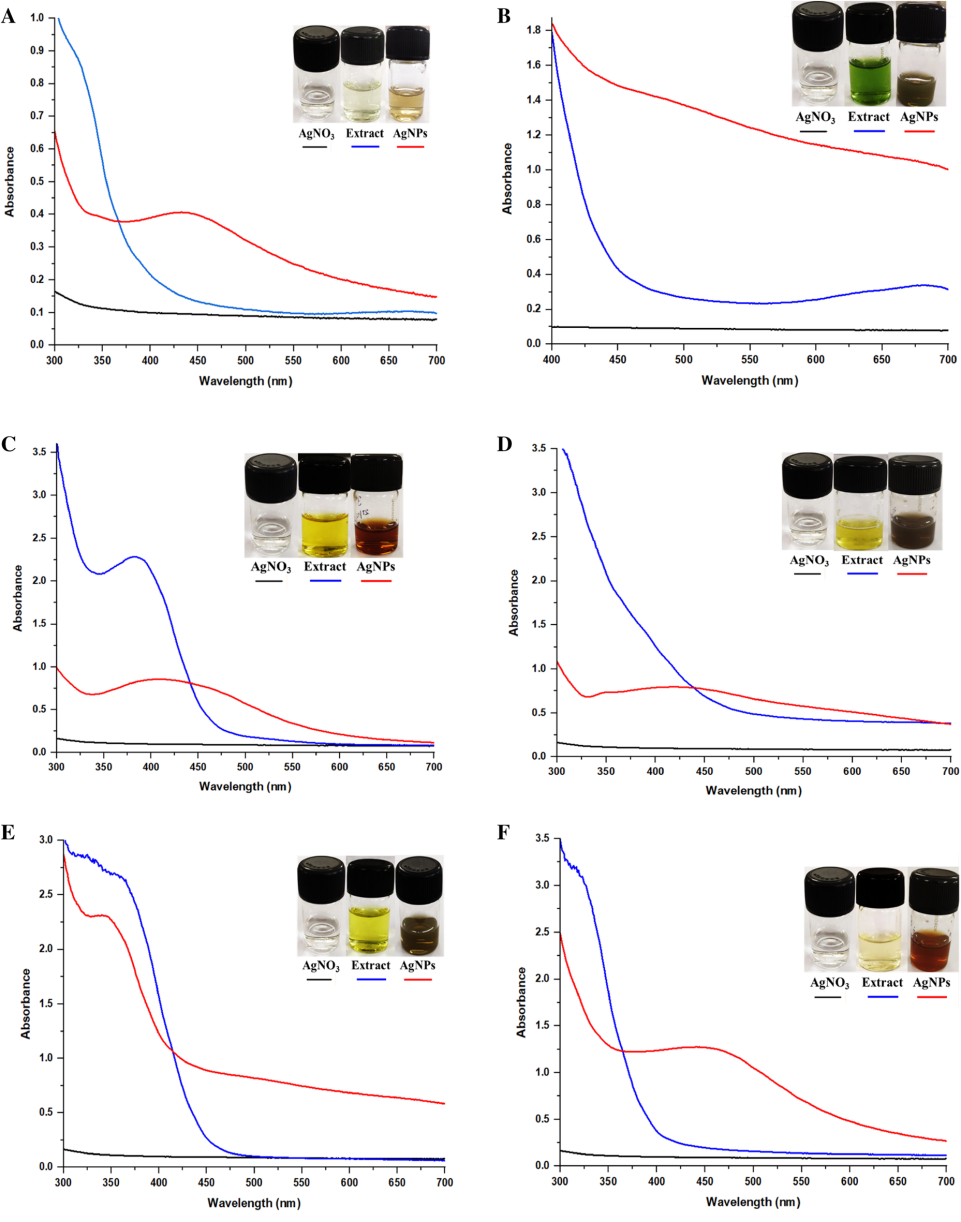

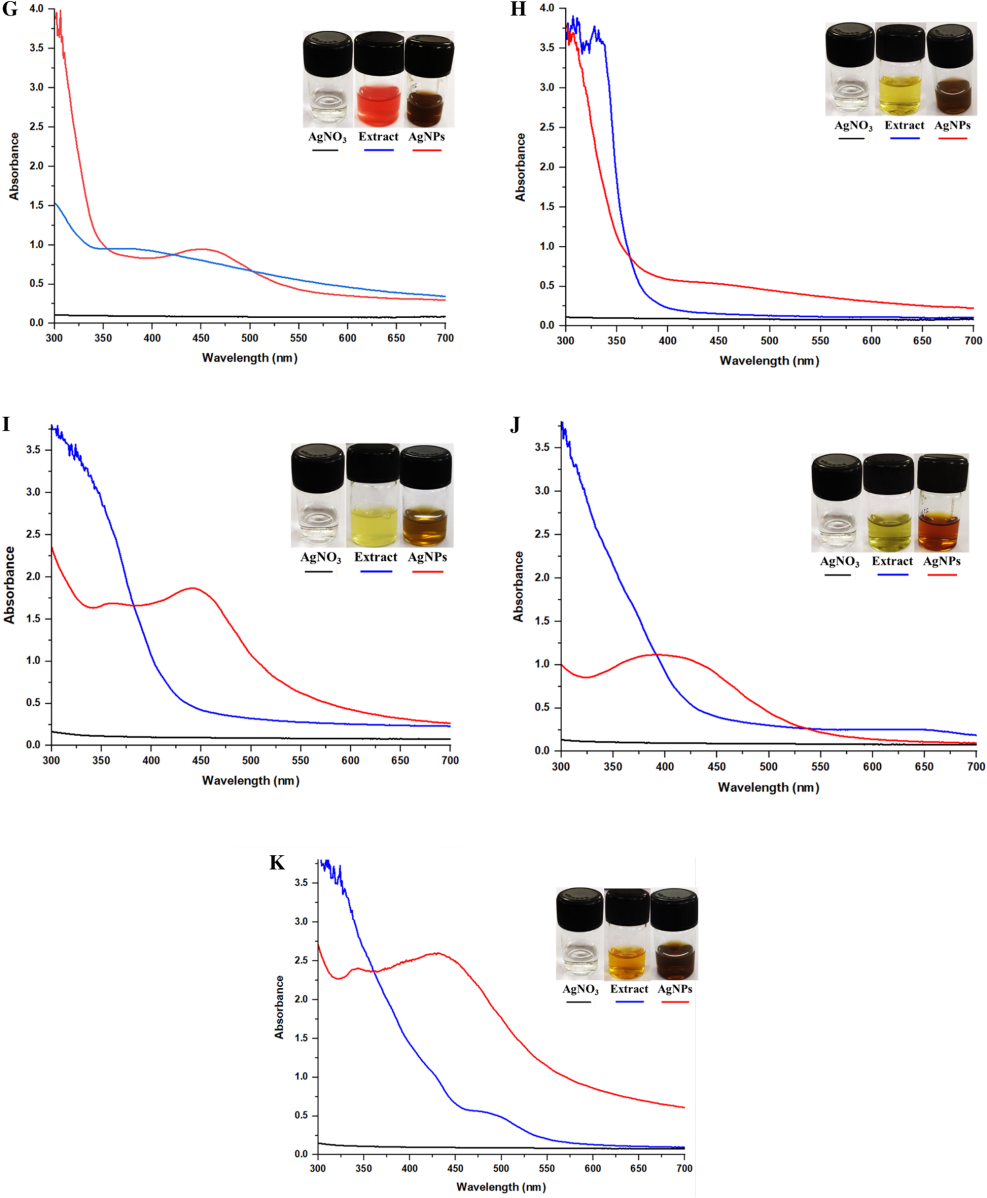
Fabrication of green synthesized silver nanoparticles using UV–visible spectroscopy. (**A**) *Jasminum* (Chameli) flower, (**B**) *Jasminum* (Chameli) leaves, (**C**) *Catharanthus roseus* (Sadabahar) flower, (**D**) *C. roseus* (Sadabahar) leaves, (**E**) *Cascabela thevetia* (Kaner) flower, (**F**) *C. thevetia* (Kaner) leaves, (**G**) *Royal poinciana* (Gulmohar) flower, (**H**) *R. poinciana* (Gulmohar leaves), (**I**) Cycas, (**J**) *Hibiscus rosa-sinensis* (Gudahal) leaves, (**K**) *H. rosa-sinensis* (Gudahal) flower.

All the selected plant extracts were screened on the basis of their potency to reduce the Ag^+^ (monovalent silver) to Ag^0^ (elemental silver). The change from AgNO_3_ to silver nanoparticles (AgNPs) resulting in change in color from yellow to reddish brown was considered as the positive response for the formation of AgNPs. JF-NPs, JL-NPs, CF-NPs, CL-NPs, CtF-NPs, CtL-NPs, RF-NPs, RL-NPs, HF-NPs, HL-NPs and Cy-NPs were synthesized and stabilized in 10 min (445 nm), 30 min (400 nm), 5 min (384 nm), 7 min (450), 25 min (340 nm), 14 h (440 nm), 12 h (450 nm), 3 min (430 nm), 2 min (450 nm), 8 min (412 nm) and 4 min (430 nm), respectively. Comparative description of LSPR peaks of AgNPs is presented in Fig. 1. The complete reduction of the Ag^+^ metal ion by the reducing sources present in the extract was measured as the stabilizing time.

**Figure 2:**
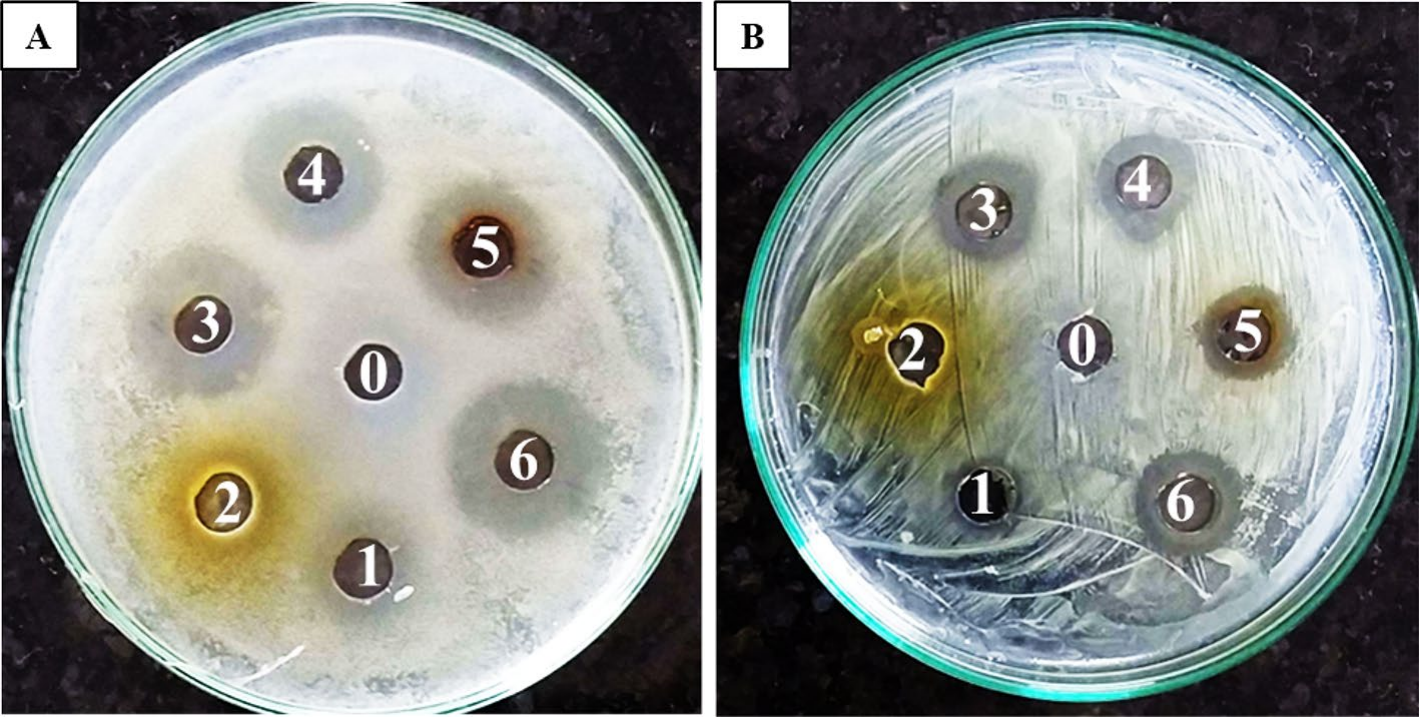
Screening of plant extract for anti-bacterial action. Effect of green synthesized AgNPs from different plant extracts on (**A**) *B. cereus* and (**B**) *B. pumilus*, where (1) Cycas, (2) *R. poinciana* leaves, (3) *C. roseus* flower, (4) *C. roseus* leaves, (5) *H. rosa-sinensis* leaves, (6) *H. rosa-sinensis* flower, (0) Control (AgNO_3_).

Furthermore, the plant extracts that took less time in the formation of AgNPs such as Cy-NP, RL-NPs, CF-NPs, CL-NPs, HF-NP and HL-NP were further assessed for their antibacterial properties against *B. cereus* and *B. pumilus*, in terms of zone of inhibition (mm) (Fig. 2) where, Cy-NP showed 13 mm; 8 mm, CF-NPs showed 21 mm; 15 mm, CL-NPs showed 20 mm; 15 mm, HF-NPs showed 21 mm; 15 mm and HL-NPs showed 19 mm; 14 mm, respectively, whereas no antibacterial activity was observed in case of RL-NPs (*R. poinciana* leaves). Based on LSPR and antibacterial assay the AgNPs formed from *H. rosa-sinensis* flower was selected for the further studies. HF-AgNPs displayed characteristic LSPR peak at 450 nm.

### Response surface methodology: parametric optimization of biosynthesis of AgNPs

**Figure 3:**
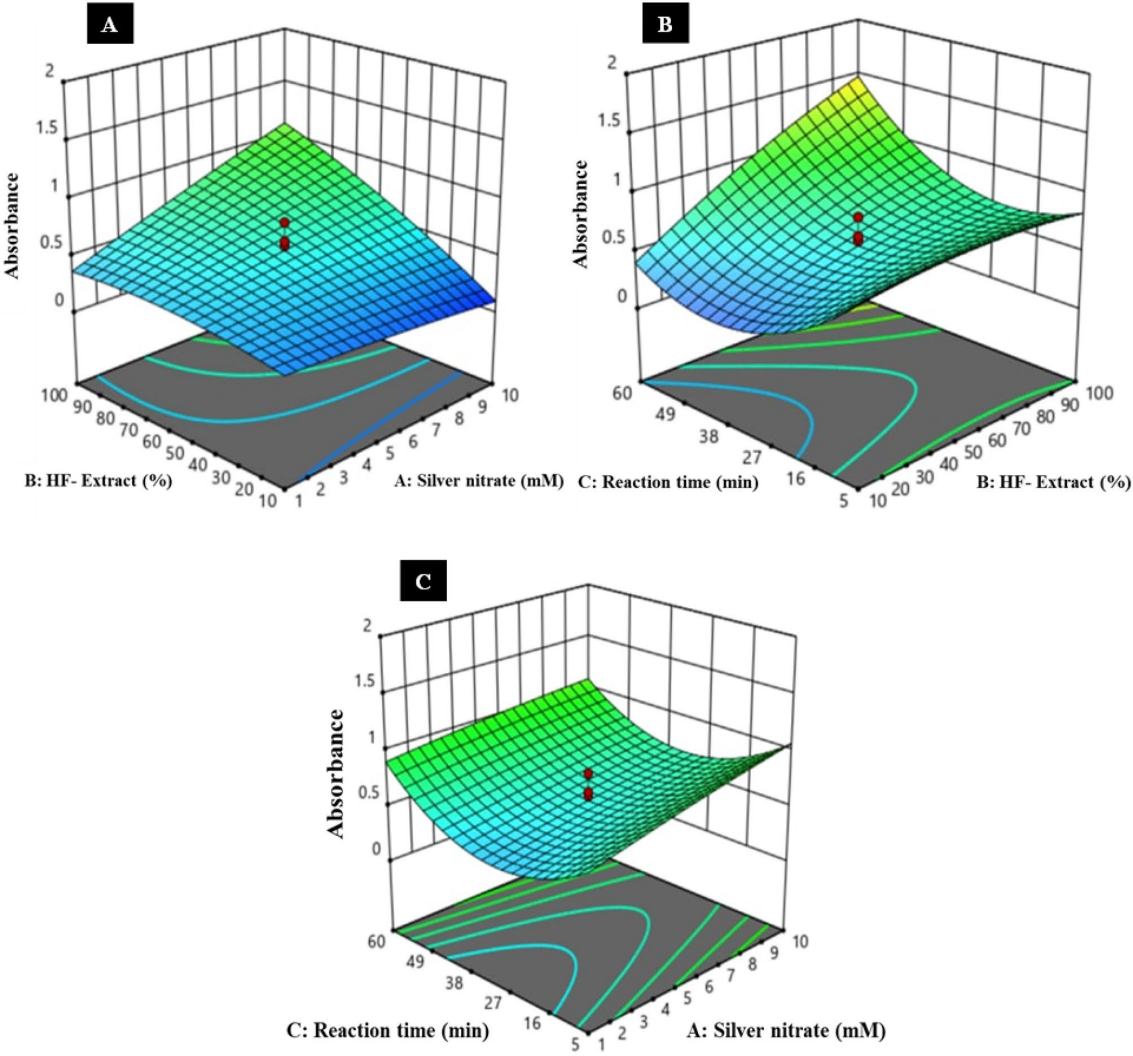
Response surface three dimensional optimization plots showing the interactive effects of three independent variables were recorded by measuring the absorbance at 450 nm, (**A**) Silver nitrate concentration (mM) and HF-Extract (%), (**B**) HF-Extract (%) and reaction time (min) (**C**) Silver nitrate concentration (mM) and reaction time (min).

The synthesis of biogenic HF-AgNPs was visually observed by the change in coloration of the reaction mixture to brown/dark brown, whereas no such color change was noticed in the control (HF-extract only). The interactive effects of three independent variables were investigated by the Central Composite Design (CCD) method. The confirmation of the formation of AgNPs was analyzed by taking absorbance at 450 nm, indicated the trial response of all the 18 distinct classes of experimental data and the collaborative relationship of the tested variables were recorded in terms of coded values (Table 1). From the maximum multinomial model point, the three individual variables were determined to have attained their maximum and ideal values. The silver nitrate concentration reached 5.5 mM, the extract concentration was at 55%, and the reaction time was set at 32.5 min. Based on these values, the predicted absorbance at 450 nm was expected to be 0.55. However, it is important to note that the actual investigational values differed from these predicted values. In addition, 0.8, 0.40, 0.48, 0.59, 0.37 and 0.63 absorbance (450 nm) data was observed in the run numbers 1, 6, 13, 14, 16, and 17, respectively (Table 1). The obtained p value of quadratic model is 0.0007 which signifies that the models are considerable and there is a numerically significant correlation among the examined variables and the measured response. Moreover, the extent of the suitable degree for the obtained model is expressed in terms of R^2^ = 0.935 (determination coefficient), which indicated that 93.56% of variation is because of independent variables in the biogenesis of AgNPs whereas about 6.4% of the disparities in the responses measured are not explicated by the model. Figure 3 presents the collaborative effects of studied variables for obtaining the highest and optimum level of the tested response, namely absorbance as assessed by 3D response and contour plots. 24.21 was found to be the F-value of the obtained model for the maximum absorbance at 450 nm with p-values less than 0.005 was found, signifying that the model stated is considerable and significant.

**Table 1:** Experimental central composite design matrix (CCD) of RSM and the investigational responses were recorded measuring the absorbance at 450 nm. Data points indicate the average of triplicate values ± standard deviation.

Run	A-Silver nitrate (mM)	B-Extract (%)	C-Reaction time (min)	Absorbance (450 nm)	
				Actual	Predicted
**1**	**5.5**	**55**	**32.5**	**0.8 ± 0.1**	**0.55**
2	5.5	55	78.74	1.69 ± 0.5	1.78
3	10	100	60	1.89 ± 0.6	1.78
4	1	10	60	0.49 ± 1.4	0.48
5	5.5	55	13.74	1.83 ± 0.05	1.58
**6**	**5.5**	**55**	**32.5**	**0.40 ± 0.05**	**0.55**
7	10	10	5	0.64 ± 5.2	0.74
8	1	100	60	1.12 ± 0.09	1.14
9	5.5	20.68	32.5	0.03 ± 2.4	0.11
10	10	10	60	0.23 ± 0.05	0.24
11	1	10	5	0.60 ± 4.2	0.82
12	10	100	5	1.15 ± 0.07	1.23
**13**	**5.5**	**55**	**32.5**	**0.48 ± 0.16**	**0.55**
**14**	**5.5**	**55**	**32.5**	**0.59 ± 0.15**	**0.55**
15	1	100	5	0.29 ± 0.04	0.39
**16**	**5.5**	**55**	**32.5**	**0.37 ± 0.03**	**0.55**
**17**	**5.5**	**55**	**32.5**	**0.63 ± 0.08**	**0.55**
18	2.06	55	32.5	0.41 ± 0.12	0.26

Significant values are in bold.

### Characterization of immobilized keratinase

#### Effect of temperature and pH on KNC

**Figure 4:**
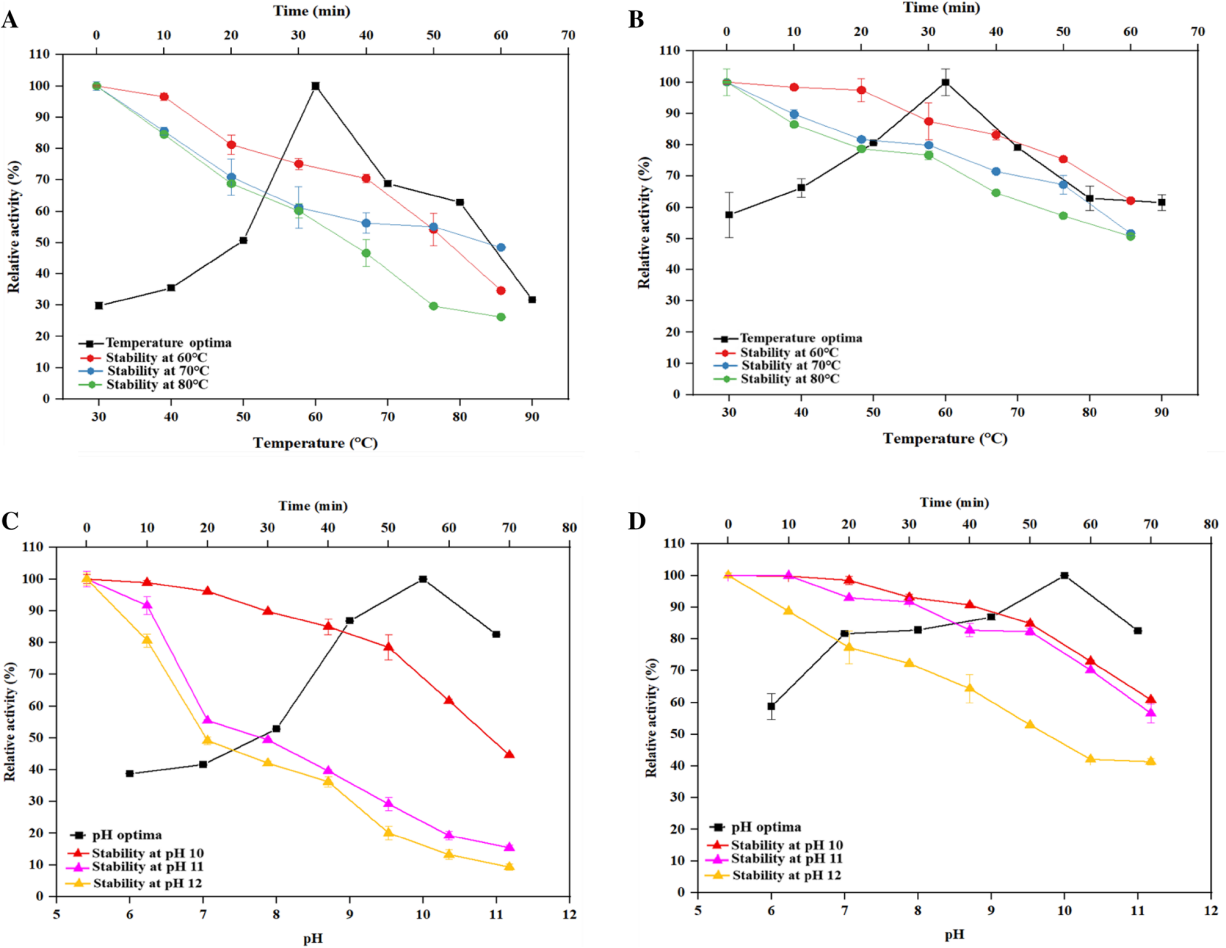
The operational profile of free and immobilized keratinase (*B. velezensis* NCIM 5802) in green synthesized HF-AgNPs. (**A**) temperature optima and thermostability of free keratinase, (**B**) temperature optima and thermostability of immobilized keratinase, (**C**) pH optima and stability of free keratinase, (**D**) pH optima and stability of immobilized keratinase. Values represent mean of three independent replicates ± SD.

The enzyme profile showed the temperature optima for both free keratinase & KNC was at 60 °C and they remained stable at 60 °C while their half-life was reached at 50 min (54.20%) and at 60 min (52.15%), respectively (Fig. 4A,B). The data stated in Fig. 4C,D indicates a wide pH activity range with an optimum at 10.0. As presented in Fig. 4C,D the free keratinase and KNC at pH 10.0, up to 60 min retained 60% and 72% of relative enzyme activity, respectively signifying their stability and tolerance for high pH. However, half of the relative activity of free keratinase was lost in 30 min (49.4%) at pH 11.0 whereas the KNC was highly stable, and its half-life reached at 80 min (49.44%), respectively.

#### Effect of additives (metal ions, detergents, solvents, surfactants, activators, and inhibitors) on KNC

The effect of various additives, such as metal ions, solvents, detergents, and modulators, on the activity of free keratinase and KNC is summarized in Table 2. The presence of Ca^2+^ and Mg^2+^ resulted in a positive effect on both free keratinase and KNC, with a significant increase in the keratinase activity. Specifically, Ca^2+^ at 5 mM concentration enhanced the activity of free keratinase and KNC by 80.5% and 105%, respectively, while Mg^2+^ at 1 mM concentration led to a noticeable increase of 205% and 223% in free keratinase and KNC, respectively. Similarly, Zn^2+^ at 1 mM concentration and Fe^2+^ at 20 mM concentration enhanced KNC activity by 61% and 77% respectively. However, increasing Zn^2+^ concentration beyond 1 mM had an inhibitory effect on both, whereas KNC showed a remarkable increase in activity even at high concentrations (upto 20 mM) of Fe^2+^. Inhibition of free keratinase was observed in the presence of Hg^2+^ and NH_4_^+^ at 1 mM concentration, while KNC retained around 79% and 101% of its relative activity.

**Table 2:** Effect of various metal ions, inhibitors, reducing agents, and solvents on the activity of *B. velezensis* NCIM 5802 free and keratinase AgNP nanocomplex (KNC). Values represent the average of three replicates ± standard deviation.

Residual activity of keratinase % (mean ± SD)										
Tested	Metal ions at different concentration									
	1 mM		5 mM		10 mM		15 mM		20 mM	
	Free Keratinase	KNC	Free Keratinase	KNC	Free Keratinase	KNC	Free Keratinase	KNC	Free Keratinase	KNC
Control	100 ± 0.1	100 ± 0.4	100 ± 0.1	100 ± 0.6	100 ± 0.05	100 ± 1.05	100 ± 0.1	100 ± 1.4	100 ± 0.1	100 ± 0.1
Cu^2+^	89.1 ± 2.1	99.7 ± 0.7	74.2 ± 1.6	101 ± 0.5	59 ± 2.3	120 ± 0.8	54 ± 1.7	132 ± 0.3	30.5 ± 1.3	128 ± 0.6
K^+^	72.6 ± 2.8	86.5 ± 0.5	66.2 ± 2.5	76.7 ± 3.2	62 ± 0.6	80.5 ± 0.4	55 ± 2.1	67 ± 4.7	47 ± 2.3	48.9 ± 0.4
Na^+^	85 ± 0.4	111.7 ± 0.8	75.3 ± 1.5	99.7 ± 2.1	72 ± 2.7	94.5 ± 0.7	52 ± 0.7	75.3 ± 2.7	33 ± 0.2	73.9 ± 0.2
Ca^2+^	154 ± 0.1	162.8 ± 0.8	180.5 ± 0.14	205 ± 0.8	131 ± 0.25	182 ± 4.1	122.5 ± 0.5	181 ± 0.4	89.2 ± 0.8	103.2 ± 0.9
Mg^2+^	205 ± 1.5	223 ± 0.12	184.2 ± 2.4	194 ± 2.1	150 ± 0.3	166 ± 0.2	70.5 ± 1.4	130.1 ± 1.7	64 ± 1.2	158 ± 0.2
Hg^2+^	62.5 ± 1.2	79.3 ± 0.8	54 ± 1.7	61.9 ± 0.7	49 ± 1.8	52.3 ± 1.6	43 ± 0.6	41.7 ± 0.16	33 ± 0.5	38.9 ± 0.15
Zn^2+^	62.5 ± 0.7	161 ± 0.9	51.3 ± 0.3	136 ± 0.6	42 ± 0.6	122 ± 0.7	40 ± 0.02	104.6 ± 0.9	24.0 ± 4.7	83 ± 0.4
Fe^2+^	57.6 ± 1.2	112 ± 0.4	44.5 ± 0.6	127 ± 0.1	121 ± 1.2	130 ± 0.4	25 ± 0.5	153 ± 0.6	19 ± 0.7	177 ± 0.4
NH_4_^+^	72.5 ± 0.6	101.8 ± 0.9	66.2 ± 0.7	84.9 ± 0.7	58.6 ± 0.4	65.8 ± 0.7	40.5 ± 0.7	62.5 ± 0.7	18 ± 1.4	35.6 ± 0.9

	Activators/inhibitors at concentration									
	1 mM		5 mM		10 mM		15 mM		20 mM	
	Free Keratinase	KNC	Free Keratinase	KNC	Free Keratinase	KNC	Free Keratinase	KNC	Free Keratinase	KNC
Control	100 ± 0.1	100 ± 0.4	100 ± 0.12	100 ± 0.6	100 ± 0.5	100 ± 1.05	100 ± 0.1	100 ± 1.4	100 ± 0.1	100 ± 0.1
Urea	35.4 ± 1.5	143.5 ± 2.3	41.2 ± 2.4	105.3 ± 0.7	16.3 ± 0.1	95.2 ± 0.4	11.9 ± 0.5	72.5 ± 0.5	10.3 ± 1.1	72 ± 1.2
EDTA	52.7 ± 2.8	87.2 ± 0.2	51.4 ± 0.52	84.5 ± 0.5	40 ± 0.12	78 ± 0.2	31.9 ± 2.2	71.9 ± 2.7	32.1 ± 1.2	35.1 ± 1.2
PMSF	7.8 ± 1.18	28.2 ± 0.18	8.6 ± 0.5	18.6 ± 0.6	6.2 ± 0.13	15.2 ± 0.3	6.2 ± 0.7	10.2 ± 0.8	5.3 ± 0.15	9.6 ± 0.5

	Detergents, surfactants, and solvents at concentration									
	0.1%		0.5%		1%		2%		5%	
	Free Keratinase	KNC	Free Keratinase	KNC	Free Keratinase	KNC	Free Keratinase	KNC	Free Keratinase	KNC
Control	100 ± 0.1	100 ± 0.4	100 ± 0.12	100 ± 0.6	100 ± 0.05	100 ± 1.05	100 ± 0.1	100 ± 1.4	100 ± 0.1	100 ± 0.1
Triton X 100	33.2 ± 0.4	268.2 ± 0.4	75 ± 0.4	243 ± 0.14	70.5 ± 2.6	219.7 ± 0.4	45 ± 0.6	158 ± 0.7	40 ± 2.1	137 ± 0.7
SDS	34.3 ± 0.3	123.1 ± 0.4	40.7 ± 0.4	196 ± 0.2	40.3 ± 0.1	375.3 ± 1.1	28.4 ± 0.9	165.1 ± 0.9	20.8 ± 3.2	57.5 ± 4.6
Methanol	29.2 ± 1.14	174 ± 1.5	25.7 ± 0.7	160 ± 0.7	21 ± 0.12	114 ± 0.2	13.8 ± 0.7	86.8 ± 0.4	11.4 ± 0.8	75 ± 1.8
Chloroform	25.1 ± 0.2	131 ± 0.9	39.7 ± 0.3	128.5 ± 0.8	22.7 ± 0.5	120.5 ± 0.4	12.4 ± 3.4	103 ± 0.5	10.2 ± 0.5	92 ± 0.3
Propanol	62.1 ± 1.7	117.8 ± 0.2	45.1 ± 0.4	104.2 ± 0.6	38.6 ± 0.7	100 ± 0.8	20.2 ± 1.4	82 ± 0.3	18 ± 1.6	74 ± 0.8

The effect of activators and inhibitors on free keratinase and KNC showed enhanced keratinase activity in case of immobilized nanobioconjuate. For example, at 1 mM concentration of urea, KNC demonstrated significantly higher activity (143.5%) compared to free keratinase (35.4%), suggesting that urea acts as an activator for KNC. Similar trends were observed with EDTA, where KNC had higher relative activity (87.2%) compared to free keratinase (52.7%). However, with increasing concentration, the inhibitory effect became more pronounced for both free keratinase and KNC. Conversely, PMSF showed a considerable loss of activity on both free keratinase (5.3%) and KNC (28%) at the same concentration. Notably, KNC exhibited significant enhancement in activity when treated with 0.1% of Triton X 100, methanol, chloroform, and propanol, with the increase in 268.2%, 174%, 131%, and 117.8% respectively, while free keratinase activity decreased by 33.2%, 29.2%, 25.1%, and 62.1%. Additionally, SDS increased the relative activity of KNC by 375.3% at concentrations up to 1%, though it effectively decreased the activity of free keratinase.

### Multi-scale characterization of HF-AgNPs and keratinase-enzyme nanocomplex (KNC)

#### Time dependent fabrication kinetics: UV–visible analysis

**Figure 5:**
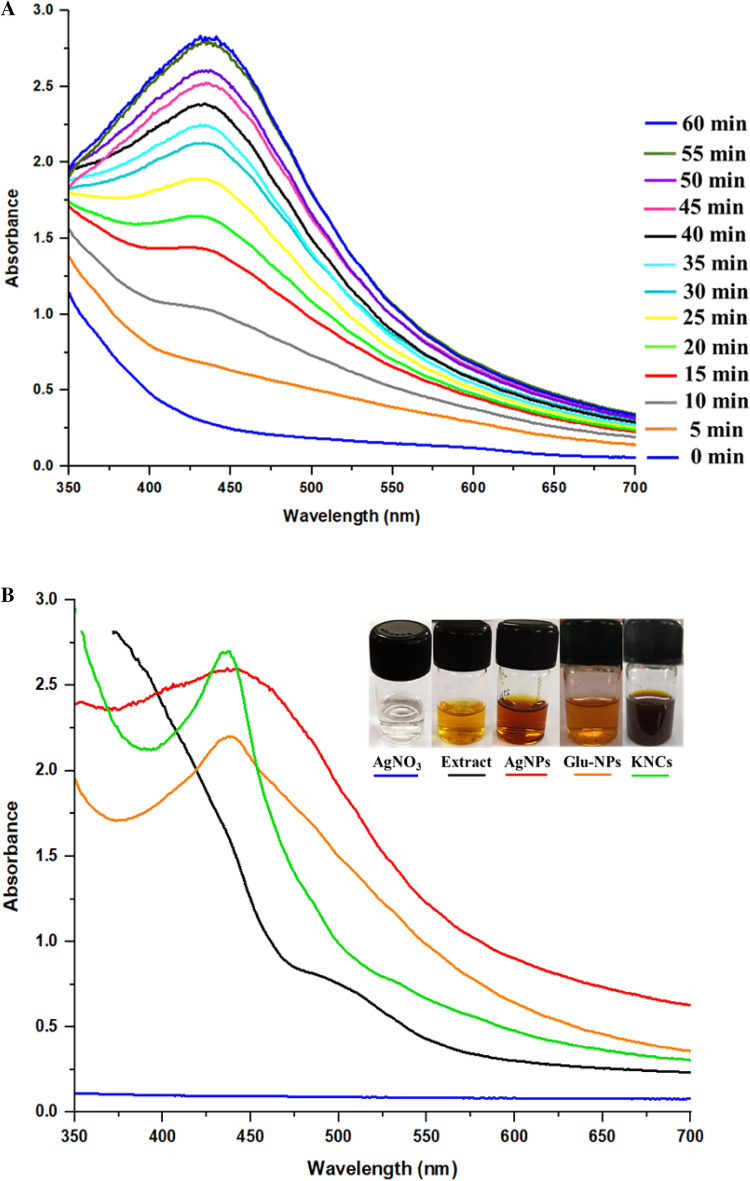
(**A**) Time dependent fabrication kinetics of biogenic AgNPs using *Hibiscus rosa-sinensis* (HF) flower extracts and its Surface Plasmon Resonance (SPR) at regular time intervals. (**B**) Surface plasmon resonance of AgNPs and keratinase-nanocomplex using UV–visible spectroscopy. AgNO_3_ (blue), *H. rosa-sinensis* flower extract (black), AgNPs (red), Glutaraldehyde activated AgNPs (orange), keratinase immobilized AgNPs (green).

UV–visible spectroscopy explains various aspects regarding the formation of nanoparticles including the size of nanoparticles along with the topographical properties of biosynthesized AgNPs. The fabrication of AgNPs was validated by a broad peak from 440 to 450 nm (Fig. 5A). The fabrication of AgNPs was distinctly visualized as the intensity and sharpness of the peak increased with respect to time (from 0 to 60 min), also the stability i.e., complete reduction of silver ion and stabilization in the formation of nanoparticles was attained after 60 min of incubation time of AgNO_3_ with reactants (plant extract).

#### Localized surface plasmon resonance (LSPR) of AgNPs and enzyme-nanocomplex

The fabrication of AgNPs from plant extract was assessed by the LSPR peaks and its comparison with the SPR peaks of glutaraldehyde activated nanoparticles (Glu-NPs) and keratinase immobilized nanoparticles (KNC) where, plant extract and AgNO_3_ were taken as a control (Fig. 5B). A wider LSPR peak from 440 to 450 nm was observed whereas a blue shift at 440 nm was observed in synthesized NPs, when subjected to surface activation (Glu-NPs) and after keratinase immobilization (KNC).

#### Hydrodynamic size analysis

Average hydrodynamic size analysis was performed to ascertain the particle size of biosynthesized AgNPs and their distribution in the solution. The typical particle size of HF-AgNPs and KNC were observed as 96.4 nm and 1225 nm with polydispersity index (PDI) of 0.291 and 0.701, respectively. Moreover, the average hydrodynamic size of HF-AgNPs was found to be 158.8 nm, while KNC showed a significantly larger average size of 6714.6 nm. The considerable increase in size for KNC may be attributed to the aggregation of the immobilized enzyme, as illustrated in Supplementary Fig. S1.

#### Transmission electron microscopic analysis of AgNPs and KNC

**Figure 6:**
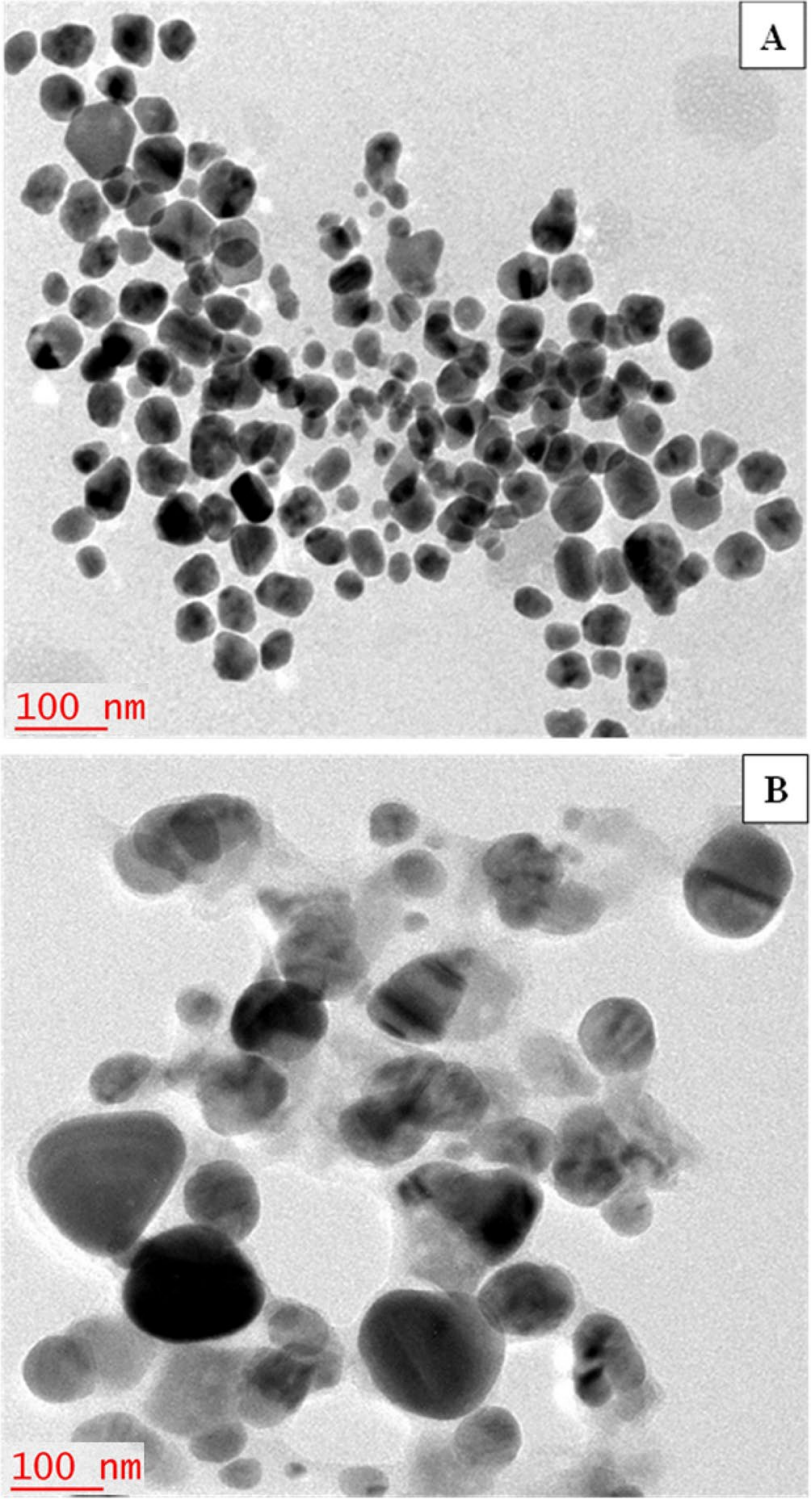
Structural analysis of green synthesizsed silver nanoparticles through transmission electron microscope. (**A**) HF-AgNPs, (**B**) Keratinase immobilized HF-AgNPs (KNC).

The morphological characteristics of AgNPs and KNC was studied by Transmission electron microscopy with the magnification of 10,000 × which revealed that the nanoparticles as well as keratinase nanocomplex (KNC) were well separated from each other and of spherical to pseudospherical in shape (Fig. 6).

#### Fourier-transform infrared (FTIR) spectroscopic analysis

**Figure 7:**
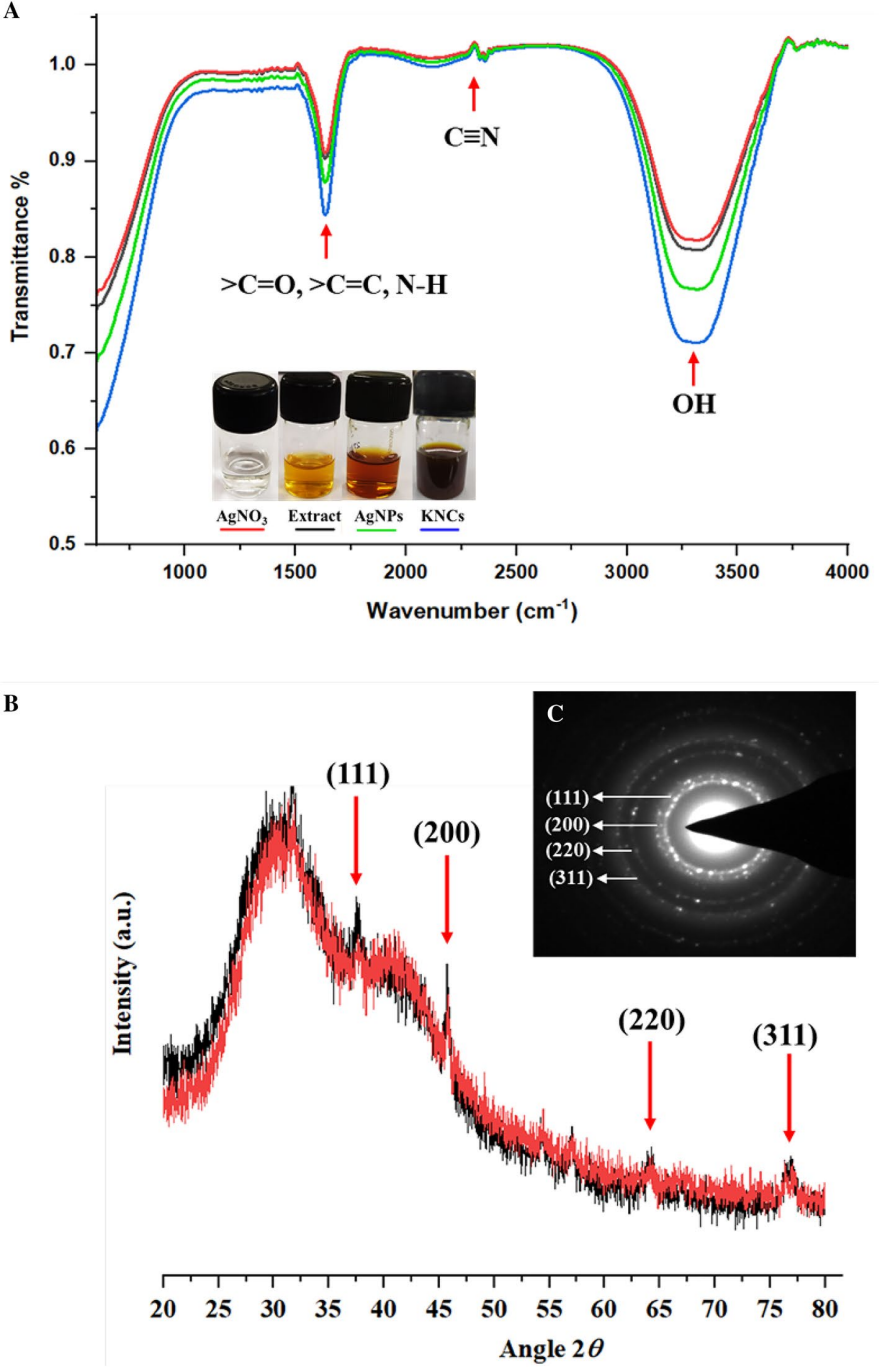
(**A**) Functional group analysis and structural changes of AgNP and enzyme-nanocomplex using ATR-FTIR. AgNO_3_ (red), *H. rosa-sinensis* flower extract (black), HF-AgNPs (green), keratinase immobilized AgNPs (blue). (**B**) X-ray diffraction patterns of the AgNPs (red) and keratinase immobilized AgNPs (black). (**C**) SAED pattern of the KNCs.

The FTIR spectra of AgNO_3_, HF-Extract, HF-AgNPs, and KNC were analyzed to ascertain the presence of potential reducing agents that play a vital role in capping and reducing silver ions into nanoparticles. The results indicated the presence of a characteristic absorption peak at 3420 cm^−1^, which signifies the presence of O–H stretch. This peak is primarily associated with polyhydroxy compounds, such as alcohols and phenolic functional groups (such as reducing sugars and phenolics), which are involved in the reduction process. Additionally, an intense stretching peak at 2340 cm^−1^ corresponds to the aldehyde C–H band, while sharp peaks at 2310 cm^−1^ indicated the presence of C≡N stretching. Weak intensity bands observed at 1480–1470 cm^−1^ suggested symmetric N–H stretching of the amine group. Furthermore, a distinctive peak at 1635 cm^−1^ can be assigned to > C=O, N–H, and > C=C stretching (Fig. 7A).

#### X-ray diffraction (XRD) analysis of green synthesized AgNPs and KNC

XRD analysis was conducted to investigate the crystal structure of HF-AgNPs and KNC. Figure 7B illustrates the XRD pattern of both HF-AgNPs and keratinase immobilized nanoparticles. The crystallite sizes (DSE) of HF-AgNPs and KNC were determined through this analysis. The major diffraction peaks at 2*θ* =  ~ 37.5, 46, 65 and 77° coinciding with (111), (200), (220) and (311) planes were employed in samples of both HF-AgNPs and KNC for the assessment of crystallite sizes.

### Applications of green synthesized HF-AgNPs

#### Antibacterial assay

**Figure 8:**
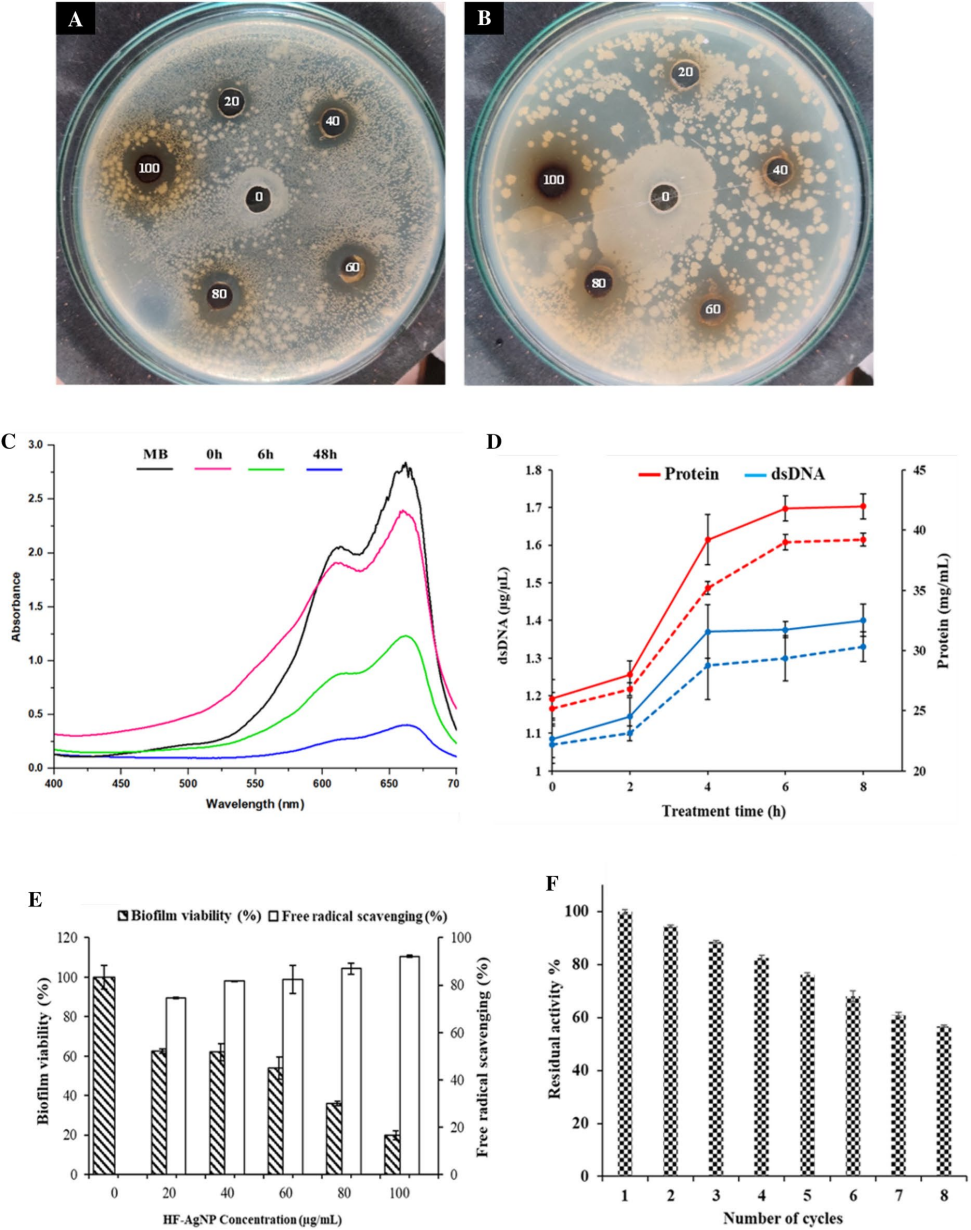
Application of green synthesized biogenic silver nanoparticles. Anti-bacterial action of KNC against (**A**) *B. licheniformis* and (**B**) *B. subtilis*, (0) Control (Glutaraldehyde), (**C**) Photocatalytic dye reduction efficiency of green KNC at different time intervals from 0, 6 and 48 h, *MB* methylene blue, (**D**) Leaching of protein (red straight line) and dsDNA (blue straight line) from *B. subtilis* with respect to the time and the untreated samples were taken as a control (protein: dotted red, dsDNA: dotted blue), (**E**) Evaluation of anti-biofilm and free radical scavenging (%) potential of biogenic KNC at different concentrations (20–100 µg/mL). (**F**) Recycling of keratinase tagged HF-AgNPs for keratin hydrolysis using feather keratin (1% w/v) at 60 °C. Values represent average ± SD of three independent replicates.

KNC were analyzed for their antibacterial activity against *B. licheniformis*, and *B. subtilis* by well diffusion method and the zones of inhibition (ZOI) around each well were measured (Fig. 8A,B). Among the bacterial strain tested, KNC exhibited the highest inhibitory activity against *B. subtilis* followed by *B. licheniformis* with ZOI of 20 ± 0.11, and 17 ± 0.45 mm, respectively at 100 µg/mL of the concentration. No ZOI was produced by control (glutaraldehyde solution) signifying the inhibitory action of KNCs itself.

#### Dye scavenging

As shown in Fig. 8C, the characteristic absorption peak of methylene blue at 660 nm disappeared in 48 h of incubation, when treated with KNC. Within 1 h of the reaction incubation 28% of MB degradation was achieved and up to 90% of the dye scavenging was attained in 48 h of incubation with KNC under sunlight (Fig. 8C).

#### Leaching of DNA and proteins

The cell disruption profile of *B. subtilis* when treated with KNC displayed an enhancement in the concentrations of DNA and protein in cell-free supernatant after 6 h of treatment when compared with untreated control sample (Fig. 8D). Selective bactericidal effect of KNC against food borne microorganisms indicated their potential uses as food antimicrobics.

#### Scavenging potential: DPPH assay

The antioxidant activity present in the green synthesized KNC was evaluated to study the ability to release free radicals in the sample as shown in Fig. 8E. Higher concentration i.e., 100 μg/mL of KNC showed significant radical scavenging i.e., 92.14% in 1 h whereas, 80 μg/mL, 60 μg/mL, 40 μg/mL, 20 μg/mL showed 86.96%, 82.32%, 81.60, and 74.46%, respectively.

#### Biofilm viability

The biofilm degradation of *B. subtilis* suggested that KNC effectively reduced the viability of the cell by 30% in 18 h (Fig. 8E). Moreover, with the increase in the concentration of KNC from 20 μg/mL to 100 μg/mL the cell viability decreases i.e., 62.54%, 61.95%, 53.86%, 36.02%, and 19.85%, respectively as compared to the control where, 100% cell viability was observed.

### Keratinase‑nanocomplex recycling

Keratinase was successfully immobilized on HF-AgNPs, with an immobilization efficacy of 87.5% with glutaraldehyde as a cross–linking agent. After surface activation, the binding of keratinase to synthesized nanoparticles occurs probably due to the mechanism of surface adsorption. The keratinase nanocomplex was recycled up to 8 cycles with 56.71% residual activity (Fig. 8F), while 50.03% activity was retained till the 9th cycle. These results indicate the effectiveness of immobilization up to 8 consecutive cycles.

### Operational storage stability

Relative activity of our enzyme nanocomplex (KNC) was found to be 76.16% at 4 °C as compared to the retention of relative activity at 30 °C i.e., 42.64% after 45 days of storage.

## Discussion

In the current study, the biogenesis of silver nanoparticles from *H. rosa-sinensis* flower extract was conducted to achieve the aim of effective synthesis and production of nanoparticles in minimum time and the process optimization for the effective biogenesis of AgNPs was accomplished through statistical optimization using CCD approach. The optimal conditions of the synthesis were 5.5 mM, 55% and 32.5 min of AgNO_3_ concentration, plant extract concentration and reaction time, respectively. The results were validated in terms of enhancement in absorbance at 450 nm. In this context, the parametric optimization revealed the efficient biosynthesis of AgNPs. Our findings are in accordance with those reported by Ibrahim et al.^4^.

A follow-up of enzyme activity profile of free keratinase and immobilized keratinase (KNC) showed a broad pH working range from 6.0 to 11.0 with optimal activity at 10.0. Both free and AgNP immobilized keratinase were operating in the temperature range of 30–90 °C with an optimum at 60 °C. The pH (10.0) and temperature (60 °C) optima of the free keratinase and KNC was not changed after the immobilization, although in comparison with the free keratinase it showed higher relative activity at all the specified temperatures. Various investigators have reported that the operational pH of most of the alkaline keratinases is close to pH 10.0^45–48^. Likewise, in accordance with the previous reports, 30–80 °C was reported as the operational temperature range for the working of the keratinase^49,50^.

After pre-treatment of KNC and free keratinase at different pH ranges for 1 h, the immobilized keratinase showed 72.9% relative activity at pH 10.0 up to 60 min, whereas activity of free keratinase was decreased drastically with time, where it retained 61.68% as compared to KNC that retained 58.36% relative activity at 60 min, respectively. These results demonstrated improved stability in case of immobilized nanocomplex. These cardinal values were in close agreement with the studies on immobilized keratinases as reported^51,52^. The enhanced pH stability of the KNC may be attributed to the structural stability of the keratinase after immobilization on AgNPs. Further, the effect of the temperature was more pronounced in case of free keratinase than the KNC, as the nanocomplex was more stable at 60 °C and retained more than 50% of its residual activity for 60 min^51–53^. This enhanced stability of KNC at elevated temperatures for longer time duration might be a result of the cross linking of the enzyme to the matrix, which prevents them from thermal denaturation. These cardinal values evidently proved that the keratinase nanocomplex had higher stability towards pH and temperature, indicating its potential applications^44^ in valorization of keratinic waste.

The free and immobilized keratinase activity was evidently enhanced in the presence of Ca^2+^ and Mg^2+^ ions indicating the role of metal ions in the functioning of the keratinase^37,54,55^. Presence of other metal ions such as, Cu^+^, K^+^, Na^+^, Zn^2+^, Fe^2+^, and NH_4_^+^ drastically inhibited the keratinase activity of the free keratinase whereas, significant endurance was observed in case of KNC, suggested that the divalent metal ion(s) are responsible for the maintenance of the active conformation of the enzyme and thus enhance the keratinolytic activity of the enzyme. Likewise, a significant decrease in the enzyme activity of both free keratinase and KNC in the presence of Hg^2+^ suggested presence of a cysteine residue at the active site^56^. Strong inhibition of the free keratinase by PMSF and EDTA reduced the activity by 8.6% and 52.7% indicating the enzyme to be a metallo-serine protease. However, KNC retained 28.2% and 87.2% activity indicating better endurance due to immobilization. EDTA chelates the metal ion(s) present at the active site of the enzyme, indicating the need of divalent metal ion(s) for the efficient catalysis^57–59^. Our results are in accordance with several researchers demonstrating the modulatory effects of metal ions on the enzyme catalysis^56,60,61^. Accordingly, with this knowledge the conditions of keratinase application may be manipulated to achieve favorable results.

Furthermore, the solvents, surfactants, and reducing agents were also found to be inhibitory for free keratinase^61–63^, whereas significant enhancement in keratinase activity was noticed in the presence of methanol, chloroform, propanol, Triton X 100, SDS, and urea by 174, 131, 117.8, 268.2, 375.3 and 143.5%, respectively (Table 2).

To understand the dynamics of the fabrication of AgNPs with respect to time, time dependent kinetics was performed where the change in color of the reactant species from colorless to dark brown was observed. This change in color is due to a specific phenomenon called as localized surface plasmon resonance (LSPR), which is the consequence of resonating oscillations created by the conduction band of the metal ions of the reactant species interacting with a certain wavelength of light^2,39^. The visible change and stabilization of the color was observed in case of HF-AgNPs at 60 min with the LSPR band from 440 to 450 nm, which confirms the formation of AgNPs^6^. Our findings are in agreement with the results reported by Choukade et al.^2^ regarding the formation of nanoparticles from two different leaf extracts, *Azadirachta indica* and *Punica granatum*, wherein the time of NPs formation and stabilization was 72 h and 9 min, respectively. Also, Ulaeto et al.^64^ synthesized the AgNPs in 24 h from the *A. indica* leaf extract. Our findings suggest the effective production of nanoparticles in comparably much less time of stabilization as compared to other reports^29,65,66^.

Also, the sequential changes in the fabrication of AgNPs, Glu-NPs and KNC were investigated and recorded in the form of LSPR peaks where, the HF-AgNP showed the SPR band from 440 to 450 nm, while Glu-NPs and KNC showed a characteristic and an intense SPR peak at 440 nm, signifying the extent of immobilization^2^. Optical properties of AgNPs, provide a great deal of information about the physical state of the sample and the aggregation of the synthesized nanoparticles^6^. Martinez-Castanon et al.^67^ observed the absorption spectrum of AgNPs between 420 and 450 nm which shifted towards blue or red light based on the increase and decrease of the particle size during the synthesis.

TEM analysis revealed the morphological characteristics of green synthesized AgNPs and KNC suggesting that the AgNPs were polydispersed and had near to spherical or pseudospherical shape whereas, the particle size of synthesized AgNPs and KNC was recorded as 96.4 and 1225 nm, respectively. These findings are in agreement with the previous reports of TEM and DLS of biogenic AgNPs^6,8^. The PDI of the AgNPs and KNC was 0.291 and 0.701, respectively. The higher PDI (> 0.50) in case of KNC indicated wide dispersal resulting in the aggregation of the nano bioconjugates^6^.

Furthermore, to uncover the functional components and their possible participation in the biogenesis of AgNPs and KNC. FTIR analysis was conducted where the changes in the intensity were used to locate the capping and stabilizing biomolecules of the green synthesized AgNPs.

O–H stretch at 3420 cm^−1^ indicated presence of polyhydroxy compounds i.e., reducing sugar and phenolics^2,40^. Also, the 2340 cm^−1^ stretch showed presence of an aldehyde C–H band, while sharp peaks at 2310 cm^−1^ indicated the existence of C≡N stretching. The presence of a weak intensity absorption bands at 1480–1470 cm^−1^ revealed the symmetric N–H stretching of amine group. A characteristic peak at 1635 cm^−1^ was accredited to > C=O, N–H and > C=C stretching^68^. The intensification of the peaks relating to free amine group and/or cysteine residues and carboxyl groups of the enzyme in KNC suggested their role in the stability in case of immobilized nanocomplex^4,69^. Our FTIR results suggested that aromatic groups and phenolic compounds of the plant extract proficiently interact with metal ions and initiate the process of reduction, capping and stabilization of synthesized AgNPs^8,70^.

X-ray diffraction pattern of green AgNPs and KNC revealed 4 intense peaks in the entire spectral range of 2*θ* values ranging from 30 to 80. These spectra corresponded to (111), (200), (220) and (311) lattice of the crystal planes^6^. This also signifies that the synthesized AgNPs had face centered cubic (FCC) structure. The XRD patterns of AgNPs and KNC are in agreement with several other reports^6,8,69,70^ that had confirmed the biosynthesis of AgNPs. The reason behind the presence of several minor peaks observed in the case of enzyme nanocomplex might be due to the presence of biomolecules or protein components which are accountable for the stabilization and integrity of the AgNPs^40^. Moreover, XRD pattern indicates that the green synthesized AgNPs were firm and crystalline in nature.

Gram-negative bacterial strain *B. licheniformis* and *B. subtilis* were employed to investigate the antimicrobial potency of formulated KNC at different concentrations, where it was found that at 100 μg/mL of KNCs, produced 20 mm and 17 mm zone of antibacterial activity, respectively. Keratinase immobilized AgNPs causes the disruption of the bacterial cell membrane by interacting with their proteins and DNA resulting in the increased cell absorption along with the DNA damage leads to the cell fatality^4^. Additionally, suggested mechanism revealed that the small sized AgNPs penetrate the cell membrane more effectively as compared to the large sized thus, disturbed the membrane permeability resulting in the cellular death. Ahmad et al.^71^ suggested that the positively charged Ag^+^ ions create electrostatic forces of attraction with a bacterial membrane which is negatively charged and hence, responsible for antibacterial ability. From the above discussion, we can conclude that the penetration of KNC in the bacterial cell membrane resulted in the damage followed by the leakage of the protein and DNA might be the plausible method of its antibacterial potency^31,72^. Thus, KNCs can effectively be utilized against wide spectrum of bacterial contamination in foods, drug delivery systems, nanogels and nanomedicine formulations.

Furthermore, light induced catalytic degradation of pigments by the application of silver nanoparticles can be used for bleaching purposes. Synthesized KNCs characteristically degraded the methylene blue dye within 48 h of incubation under sunlight. The mechanism of action behind the dye degradation is based on the formation of free radicals when keratinase tagged AgNPs and dye was incubated in the presence of light, the photon catalyzed reaction reduced the colorful dye into leuco form. A similar kind of study was carried out where methylene blue was reduced by 82.8% in 180 min after the exposure in mercury light with AgNPs^73^. Chen et al.^74^ reported that the AgNPs enhanced the photocatalytic activity by causing the charge separation of light generated e^-^ hole pairs, also, the degradation of dyes ensues significant application in remediation of environmental pollutants such as NPs derived from Ag, Au, ZnO etc. are used for wastewater treatment^75^.

AgNPs generate free radicals such as O_2_^**∙**–^, **∙**OH, O=O, HClO and H_2_O_2_^2^. According to the previous findings antioxidant activity is directly proportional to the presence of phenolic compounds and flavonoids in the plant extracts^2^. Antioxidant potential is due to the neutralization and stabilization of the functional groups of the plant extract together with the aggregation of the AgNPs with time^30^. KNCs showed higher antioxidant activity signifying the contribution of bioactive molecules of the plant extract and these findings are in accordance with the results concluded by Khorrami et al.^29^ and Kamaraj et al.^76^. High antioxidative activity of the green KNCs entails its functional and practical usage, predominantly in cosmetic, drug and food manufacturing.

Recycling is the principle purpose of enzyme immobilization. In this study, the KNC was found to be functional up to eight consecutive cycles of keratin hydrolysis, retaining more than 60% of its relative activity. Also, the shelf life of the keratinase nanocomplex after 45 days of storage was found to be 76.16% at 4 °C and 42.64% at 30 °C, respectively. The average activity of the enzyme nanocomplex was 78.40% which revealed the functional efficiency of the immobilized system and suggested no such loss in the activity due to degradation or denaturation^2,44^. Efficacy, operational stability, and shelf-life of the KNC suggested feasibility of the enzyme-nanocomplex synthesis and its application in valorization of poultry keratin.

## Conclusion

Optimized biogenesis of silver nanoparticles was achieved using *H. rosa-sinensis* flower extract using response surface methodology (RSM). Keratinase nanocomplex was immobilized onto synthesized silver nanoparticles and KNCs were optimally active at pH 10.0 and 60 ℃. Multi-scale spectroscopic analysis using UV–visible, FTIR and XRD revealed the presence of capping and stabilizing agents in the plant extract that are responsible for the formation of AgNPs. The produced AgNPs and KNCs were also characterized by time dependent kinetics, particle size analysis, and TEM suggested that the produced NPs were spherical to pseudospherical in shape with fairly small size. The green synthesized keratinase tagged AgNPs were also evaluated for their antibacterial, antiradical, biofilm mitigation and dye degradation revealing their potential applications. The results presented in this article provide newer insights for environmentally benign and cost-effective preparation of nanoparticles along with their usage in immobilization of biomolecules, biomedical, cosmetic industries and in environmental remediation.

## Supplementary Information

Supplementary Figure S1.

## Data Availability

The 16s rRNA sequences of *Bacillus* sp. NCIM 5802 were submitted to the NCBI accession number ON203026 (https://submit.ncbi.nlm.nih.gov/subs/?search=SUB11319989) and the bacterial culture is submitted to NCIM accession number NCIM-5802 (https://www.ncl-india.org/files/ncim/CatalogueDetails.aspx?NCIMNo=5802). The authors declare that all data supporting the findings of this study are available within the article.

## Abbreviations

RSM: Response surface methodology
CCD: Central composite design
ANOVA: Analysis of variance
SD: Standard deviation
KNC: Keratinase nanocomplex
AgNPs: Silver nanoparticles
EDTA: Ethylenediaminetetraacetic acid
PMSF: Phenylmethylsulfonyl fluoride
SDS: Sodium dodecyl sulfate
TEM: Transmission electron microscope
FTIR: Fourier transform infrared spectroscopy
XRD: X-ray powder diffraction
MB: Methylene blue
