# Supplementary material for: Synthesis and characterization of keratinase laden green synthesized silver nanoparticles for valorization of feather keratin
Source: Sci Rep. 2023 Jul 18;13:11608. doi: 10.1038/s41598-023-38721-6 (PMC10354033; doi:10.1038/s41598-023-38721-6)
Supplement: Supplementary file 1 — Supplementary Figure S1. [file 41598_2023_38721_MOESM1_ESM.docx]

**Supplementary Figure S1:** Hydrodynamic size analysis representing the particle size and distribution of A) biosynthesized AgNPs and B) keratinase nanocomplex (KNC).


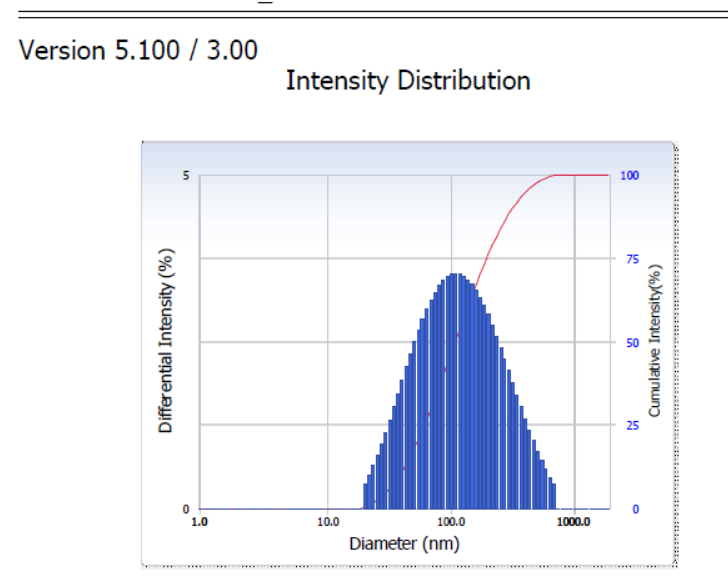

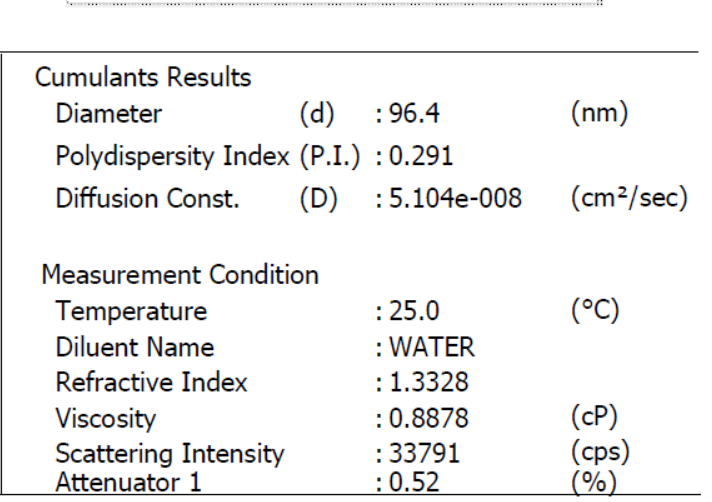

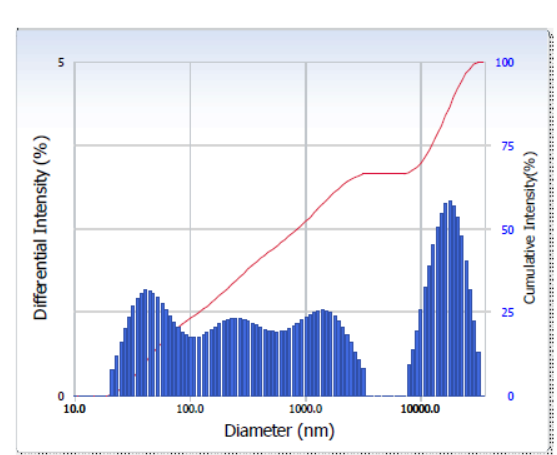

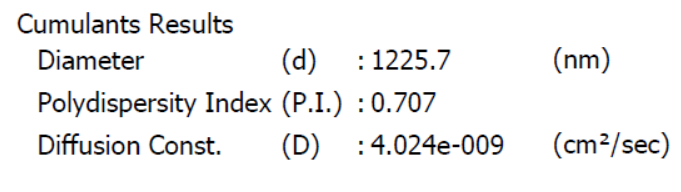


**A**

**B**
